# Normalizing brain activity across individuals using functional reference mapping

**DOI:** 10.1038/s41598-017-16913-1

**Published:** 2017-12-07

**Authors:** Eugenio Martinelli, Alja Lüdke, Piergiorgio Adamo, Martin Strauch, Corrado Di Natale, C. Giovanni Galizia

**Affiliations:** 1 Department of Electronic Engineering, University of Rome Tor Vergata, Via del Politecnico 1, 00133 Roma, Italy; 2 Neurobiology, Department of Biology, University of Konstanz, D-78457 Konstanz, Germany; 3 Present Address: Institute of Imaging & Computer Vision, RWTH Aachen University, Aachen, Germany

## Abstract

Neural activity can be mapped across individuals using brain atlases, but when spatial relationships are not equal, these techniques collapse. We map activity across individuals using *functional* registration, based on physiological responses to predetermined reference stimuli. Data from several individuals are integrated into a common multidimensional stimulus space, where dimensionality and axes are defined by these reference stimuli. We used this technique to discriminate volatile compounds with a cohort of *Drosophila* flies, by recording odor responses in receptor neurons on the flies’ antennae. We propose this technique for the development of reliable biological sensors when activity raw data cannot be calibrated. In particular, this technique will be useful for evaluating physiological measurements in natural chemosensory systems, and therefore will allow to exploit the sensitivity and selectivity of olfactory receptors present in the animal kingdom for analytical purposes.

## Introduction

Sensory systems provide animals with information about the environment with a level of efficiency that, in some cases, largely exceeds that of technological equivalents. This is certainly the case in olfaction where so-called electronic noses are still rudimentary compared to biological olfactory systems. Several attempts have been done in the past to incorporate elements of olfaction, at various level of complexity, into sensors^1^. For instance, silicon devices have been coupled with odorant binding proteins^2^ or even with olfactory receptor neurons^3^. A different approach consists in recording the physiological responses of olfactory receptor neurons in living animals. Here, experimental methods used in neuroscience, such as population calcium imaging to record functional activity patterns of olfactory neurons, generate data that can be used to discriminate among volatile compounds^4^. However, phenotypic differences affect the comparability of the recordings among different individuals. This affects analytically necessary properties such as reproducibility and stability. In artificial sensor arrays, we might place or wire the sensors with a known response profile according to their functional properties, and thus facilitate their readout. However, when using an olfactory epithelium or an insect antenna, the responses appear spatially scrambled, making it difficult to compare spatial activity patterns from one antenna to the next. In this paper, we propose a system to use the activity maps themselves to register functional responses across olfactory epithelia (antennae) in the fruit fly *Drosophila melanogaster*.

The spatial organization of neurons in the nervous system is genetically controlled to a large extent. For this reason, functional imaging data can be compared across individuals. At a large scale, brain areas are identified morphologically and their activity compared directly (e.g. “visual cortex”, “motor cortex” or “hippocampus”). But also at smaller scale, within functional areas, the geometrical arrangement of activity is conserved across individuals. In these cases, a stereotactic approach is useful. One approach uses elastic registration to match different brain images using identifiable landmarks^5,6^. The underlying assumption is that spatial arrangement of activity is faithfully represented at the interpolated locations. For example, in the mouse olfactory bulb, olfactory glomeruli are arranged in a stereotypical manner, e.g. the mouse mOR23 is always located medially^7^. However, at a small spatial scale, variability across individuals increases and interpolation based on morphological and/or functional landmarks becomes unreliable^7^. The precise location of genetically determined glomeruli is variable, with groups of a few glomeruli swapping their relative position^8^. Therefore, at fine resolution, a registration across individual brains based on geometric and spatial criteria is impossible. This lack of fine-scale registration is also present in the periphery, i.e. when we consider the precise location of identifiable olfactory neurons in the epithelium (in mammals) or on the antenna of insects, such as *Drosophila melanogaster* ^9^, also because neuronal populations are scattered in overlapping areas in these organs. Here we propose that rather than using geometry, it is possible to use neural activity itself for calibration, and perform *functional registration*.

**Figure 1:**
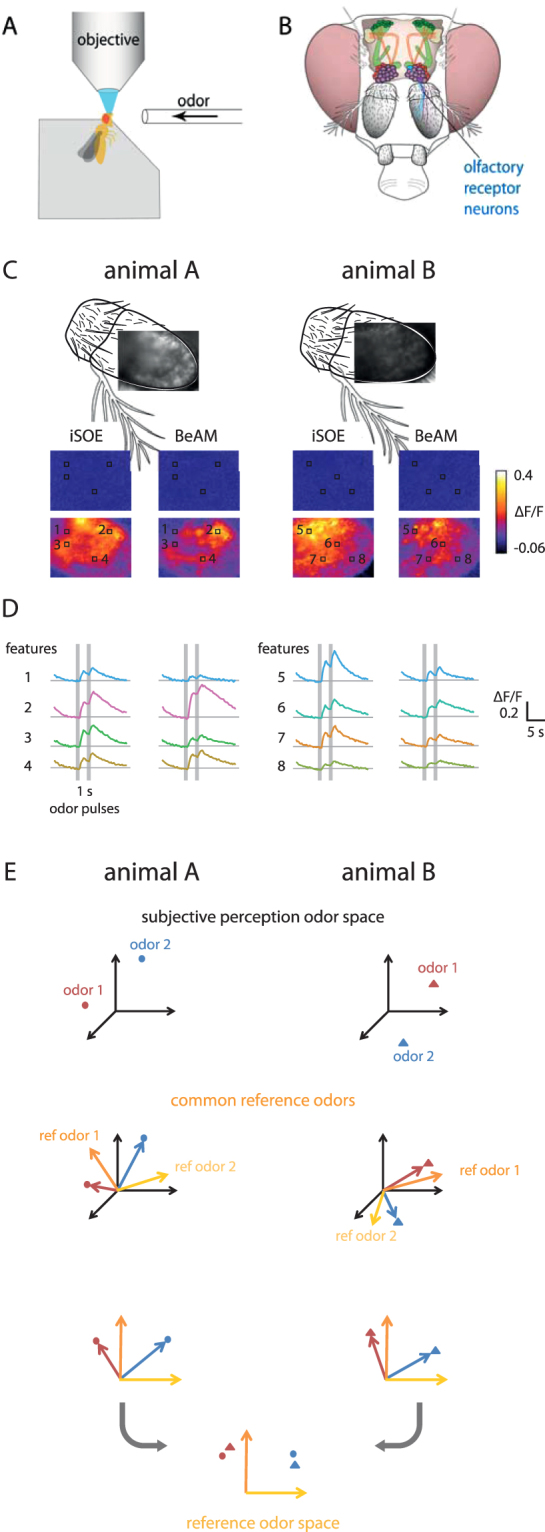
Conceptual procedure of functional reference mapping, used for *Drosophila* odorant responses **(A)** Setup for recording odorant-evoked calcium imaging data from the fly’s antenna. A living fly is fixed in a microscope (objective), and odorants are delivered to the antennae. **(B)** Schematic of a *Drosophila* head. The antennae with olfactory receptor neurons (blue) are shown. Within the brain, a schematic of the olfactory circuitry is shown, with the antennal lobe in purple, and the mushroom bodies in green. These are the brain areas that decode odour information. **(C)** Examples of calcium recordings. Top: image of the antenna with GCaMP-fluorescence, and the missing part of the antenna shown schematically. Bottom: false colour coded odorant responses to the odorants isoamyl acetate (iSOE) and benzaldehyde (BeAM), in two frames: one before odorant stimulus (upper), and one during odorant stimulus (lower). Local regions of interests (features) are shown as squares. **(D)** Calcium odorant responses for individual features from (**C**), showing the two response peaks to the two stimuli. Note that response magnitude and shape differ between antennal regions (numbered, and evidenced by colour), and for different odours (iSOE left, BeAM right, within each animal). **(E)** Idea of functional reference mapping: for each animal, two measured odorants (odour 1, odour 2) can be represented in a multidimensional space created by all feature responses (*subjective perception odour space*). When they are mapped onto a reference odour space created by *common reference odours* (yellow and orange arrows in the centre images), and rotated accordingly, they can be merged into a *reference odour space* (bottom).

We used the peripheral olfactory system of *Drosophila melanogaster* as a test case (Fig. 1A,B). Olfactory receptors are arranged along the surface of the antenna in a genetically determined way, but the small-scale arrangement of activity patches is different from animal to animal, due to genetic and experimental variability^9^. Some of these receptors exhibit a broad response range to odorants, and odours elicit combinatorial activity patterns^10^. We suggest that a linear transformation could be used to map odours within a functional reference odour space. We have previously used *Drosophila* to detect and discriminate clinically relevant odorants, e.g. from cancer cells^4^. However, since every animal is an autonomous sensorial system, the derived qualitative features could not be directly compared or averaged across different flies. Here, we introduce a virtual reference system based on functional responses, rather than spatial locations, in order to quantitatively compare brain activity across individuals.

## Results and Discussion

We expressed a genetically encoded calcium sensor in olfactory receptor neurons (ORNs) of *Drosophila melanogaster*, and recorded their responses to olfactory stimuli as a change in fluorescent signal (Fig. 1C,D). Next, we defined a multidimensional functional stimulus space based on a set of reference odours, assuming that the relative positions of (any) odorant should be the same across individuals. This is reminiscent of sensorial analysis, e.g. when describing a particular odour (say, a wine) with predefined qualitative reference axes (“subtle cedar wood, oriental earth, dried plum, minor raspberry”). Since similar molecules activate the same ORNs in different flies, the topological arrangement in a functional multidimensional space should be comparable. Thus, we adopt a set of reference odours to map the subjective perception of the signal into a space where measures from different flies are comparable (Fig. 1E).

**Figure 2:**
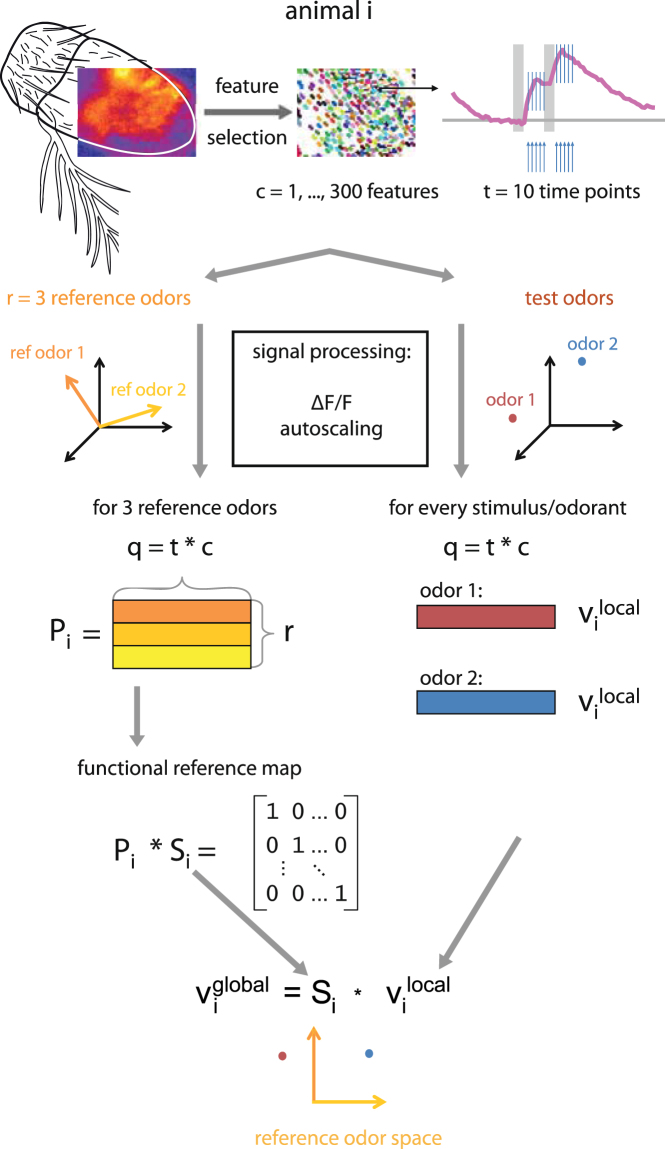
Schematic of the functional reference mapping procedure. From top to bottom: in the optical imaging recording of a fly antenna (left), *c* = *300* features are selected (centre), and for each of these features, the 10 most informative time points (*t*) are taken (responses to the first and second odorant stimulus, right). Next, signals are processed (ΔF/F, autoscaling), separately for the test odorants (right) and the *r* = *3* reference odorants (left). Each odorant response (test odour, or reference odour) gives a vector sized *q* = t * c. These are the *v*_*i*_^*local*^ for test odorants, while the *r* reference odorants are assembled to the matrix *P*_*i*_ (for this animal *i*). *P*_*i*_ is used to calculate an animal-specific *S*_*i*_, and *S*_*i*_ is used to remap each *v*_*i*_^*local*^ into *v*_*i*_^*global*^.

We recorded odorant-elicited calcium increases in ORNs on the *Drosophila* antenna by expressing GCaMP3 under the control of Orco-Gal4 (see methods). Single recordings were 20 s long, consisting of *s* = *80* frames measured at 4 Hz with two identical odorant stimuli lasting 1 s each. This arrangement recorded an odorant response in a non-adapted (1^st^ stimulus), and in a slightly adapted state (2^nd^ stimulus), maximizing odorant response information (Fig. 1D). The procedure is shown in Fig. 2: optical recordings across the insect cuticle in the antenna gave spatially structured activity maps (top left in Fig. 2). Olfactory sensilla are expressed in genetically controlled patches along the antenna^11^, generating spatial maps. However, calcium signals from the neurons are scattered by the cuticle, so that individual sensilla cannot be recognized. Therefore, signals in individual pixels represent linear combinations of responses from different receptor types, in differing weights. Furthermore, over time, responses are strong right after odorant stimulation. Therefore, we reduced the number of pixels to *c* = *300* features in order to reduce redundancy (Fig. 2, top centre). We selected *t* = *10* time points (top right) to focus on the informative time points right after stimulation. Thereafter, all data processing was done separately for the reference odours (Fig. 2, left column) and the test odours (Fig. 2, right column). Signals were processed as relative fluorescence changes (ΔF/F, autoscaling), resulting – for each odorant – in a response vector sized *q* = t * c. These we call the *v*_*i*_^*local*^ for test odorants (vector representing this odour locally in animal *i*). We assembled the *r* reference odorants (in this example *r* = *3*) into the matrix *P*_*i*_ (for animal *i*). *P*_*i*_ was then used to calculate the animal-specific transformation matrix *S*_*i*_, and *S*_*i*_ was used to remap each *v*_*i*_^*local*^ into *v*_*i*_^*global*^ (vector representing the response to this odour in animal *i* in the global odour space). In this way, odour-response vectors from different animals could be mapped into a common multidimensional reference system (Fig. 2, bottom). The information previously taken from other flies can now be integrated, allowing for taking a decision (odour categorization) based on the cumulative information from many animals. Different classifiers can be used, such as k-NN (k-nearest neighbour) or SVM (support vector machine)^12^.

**Figure 3:**
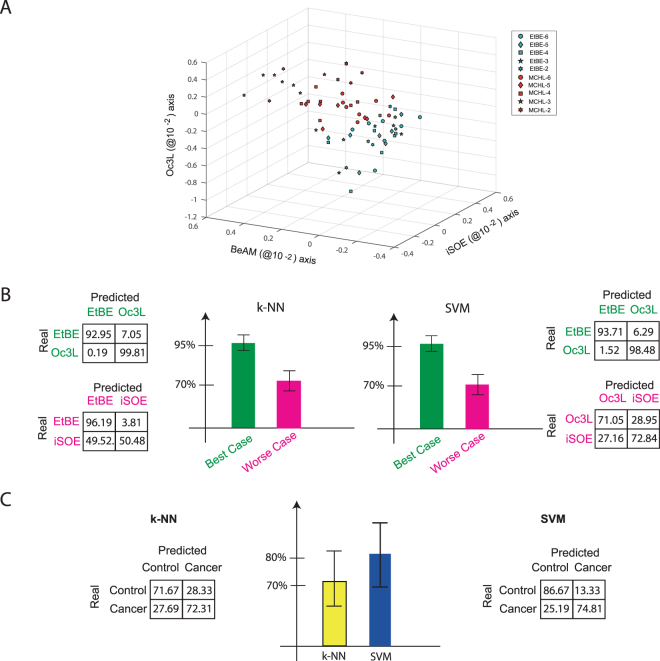
Results of the olfactory signals representation in two considered experiments. **(A)** Representation of signals of two compounds (five concentrations each) in the base of three reference compounds (Oc3L, BeAM and iSOE). **(B)** Rate of correct classification and confusion matrices obtained processing the matrix of projected signals in the best and worst representation bases, given in percentage. k-NN (left) and SVM (right) are compared as classifier algorithms. **(C)** Rate of correct classification and confusion matrix of the signals related to cancer cell odorant experiment. Classification was performed by k-NN (left) and SVM (right) algorithms.

To probe the efficacy of our method we performed two sets of experiments. In the first one, we measured five different odorants: 4-methylcyclohexanol (abbreviated to MCHL), isoamyl acetate (iSOE), benzaldehyde (BeAM), ethyl butyrate (EtBE), and 3-octanol (Oc3L) in *n* = *7 Drosophila* flies. Each odorant was given at five concentrations, in decadic steps from 10^−6^ to 10^−2^. We mapped the odorants into a common functional space as described above, and classified them using k-NN or SVM classification, in order to probe whether the same odours cluster together irrespective of the measured animal. We selected three reference odorants using only the highest and the lowest concentration (excluding the intermediate concentrations, in order to reduce the danger of overfitting), and used the remaining two (across all concentrations) as test odorants. Concentration was not a parameter in the cross-validation procedure, but used to test the robustness of the procedure: a sensor needs to be concentration invariant over several orders of magnitude in order to be useful. A sample 3-D projection of the two test odours (across all concentrations) is shown in Fig. 3A. We ran this for all $$(\begin{array}{c}5\\ 2\end{array})=10$$ odorant combinations. The best results were obtained for MCHL, iSOE and BeAM as reference odorants (k-NN classifier 96.3% accuracy, Fig. 3B; average of 35 cases with 4 training animals, and 3 testing animals each), the worst results for MCHL, BeAM and EtBE as reference odorants (accuracy of 73.3 ± 8.9% with k-NN classification). Using an SVM classifier yielded similar results, with 96.1 ± 4.1% accuracy in the best case (same odorants as for k-NN), and 71.9 ± 6.17% in the worst case (odorants EtBE, MCHL, BeAM as reference odours). The large difference between the best and the worst performance indicates that the choice of reference odours is important for this technique: only areas in stimulus space that are covered by reference odours can be mapped accurately. In the wine analogy mentioned above: if the reference axes would be “cedar wood”, “dried plum” and “rasperry”, it would be difficult to place “diesel” in the reference space. Reference odours and test odours need to be related.

In a second experimental approach, we measured nine types of odorants with *n* = *10* flies (Fig. 3C). Three different human breast cancer cell lines were used in the experiment and one immortalized, non-transformed human mammary epithelial cell line referred to as healthy control. To build the functional reference space, three reference odorants were used: propanoic acid (ProS), ethanoic acid (EtaS), 1-butanol (ButL), as well as two stimulus controls: medium (culture medium), and the solvent mineral oil (MOL). From every animal, we obtained four measurements for test odours (one healthy control and the three cancer lines), resulting in 40 measurements in total (10 animals × 4 odorant response signals). The results obtained considering all possible combination are shown as a confusion matrix with the corresponding accuracy (Fig. 3C). When using 8 animals for training, and 2 animals for testing, we found an overall accuracy of 72.0% for k-NN, and 80.7% for SVM. We have run the same classification for all cases between 5 training (and 5 testing) animals and 9 training (and 1 testing) animals: the more training animals were used, the better the classification (data not shown). These data show that the approach can also be used for complex odorant mixtures as test odors, in a reference system of pure chemicals as reference odours.

The two experiments reported here allowed us to cluster odorant responses across animals in a reliable way, by mapping them onto a functional reference space spanned by responses to known reference stimuli. We applied the technique also to complex odorant mixtures of unknown chemical identity, aiming at identifying, in a promising non-invasive way, the presence of cancer tissues (experiment 2, Fig. 3C). We consider this to be a major breakthrough, because it allows to assemble odorant responses from different animals, thus exploiting the olfactory knowledge previously acquired. However, many aspects remain to be investigated, and to be considered for particular applications. Using biological sensors implies, inherently, the necessity to deal with variability. Sensory epithelia may differ due to genetic background, to developmental changes (e.g. wearing off with age), and to plasticity (learning and/or adaptation). The strength of our approach is that much of this variability is self-correcting, since the reference odours are measured under the same system conditions as the test odours. Therefore, if the system changes by wearing off with age, the response patterns of the reference odours should shift in concordance with the response patterns of the test odours, but within the transformed odour spaces the positions of the test odours should remain constant. There will be situations, though, where this stability collapses (e.g. when a particular receptor, or a group of receptors sensitive to one of the used odours, is missing). Therefore, in future experiments, the choice of reference odorants and their concentration will need to be addressed (as a function of which odours to be classified). From a geometric point of view, the subspace spanned by the reference odorants should share as many dimensions as possible with the (unknown) subspace of the target stimuli. For example, in experiment one we used 5 odorants, three as reference and two as test odorants, and found that performance was highly dependent on the choice of which odorants where used as reference. In the olfactory epithelium, every odorant will activate a set of receptors in a combinatorial manner. If the set of receptors activated by the reference odorants is entirely disjunct from the set of receptors activated by the test odorants, then the approach presented here will fail. Similarly, at very low concentrations, odorants activate only very few or even only a single receptor type^13^. Unless this receptor is also activated by the reference odorant, it will not reliably project into the reference odour space. In a sensorial description: if all reference odorants are “fruity”, a test odour that smells “burned” will be misjudged (but might still be consistently mapped onto a reproducible location). Finally, for every application, the number of reference odorants (i.e. the dimensionality of the reference odour space) needs to be determined: more dimensions will allow for better resolution, but higher costs. These costs involve, in particular, measurement time. Using biological tissue implies that measurement time is limited by fatigue, adaptation, nutrient supply and tissue longevity; therefore, in every single situation, a compromise has to be struck between resolution (based on quantity, which increases with more measurements) and reproducibility (data quality, which decreases with more measurements). Also in biology, animals have evolved very different numbers of olfactory receptors in their genomes, affording their differences in olfactory performance.

## Conclusions

We propose that the multidimensional rotation technique presented here will allow for several new approaches into analysing physiological data. Animal olfactory cells are highly sensitive for a variety of odorants, such as medically diagnostic odorants or explosives^14^, and many offer high selectivity. Our technique will allow combining several animals to create metasensors ready for practical applications, also in situations where the individual receptors cannot be measured separately. The general nature of this technique can be also applied to biosensors and bio-electronic devices incorporating biological elements. In brain science, *functional mapping* may be used to create functional brain atlases even without morphological reference systems.

## Material and Methods

### Flies

The flies used for calcium imaging (genotype: w; P[UAS:GCaMP3]attP40/CyO; P[Orco:Gal4]) expressed the calcium reporter GCaMP3 in all Orco bearing cells (UAS-GCaMP3 flies were provided by Loren L. Looger, Howard Hughes Medical Institute, Janelia Farm Research Campus, Ashburn, Virginia, USA). Flies were kept on standard medium (100 ml contain: 0.7 g agar, 2.4 g yeast, 2.1 g sugar beet syrup, 7.1 g cornmeal, 6.7 g fructose, 1.4 ml Nipagin-10%, 0.6 ml propionic acid), at 25 °C and 60% rel. humidity, on a 12/12-hour light/dark cycle.

For calcium imaging of the olfactory receptor neurons (ORNs) on the fly’s antenna, the fly was fixed in a mounting block with soft wax around the head and the antenna was further fixed by gluing the arista to the eye or to the block and further by placing a small metal grid onto the second antennal segment. Either the right or the left antenna was recorded from.

### Odorant preparation

Odorants were purchased from Sigma (Sigma-Aldrich, Steinheim, Germany) in highest purity and diluted in 5 ml mineral oil (Sigma-Aldrich, Steinheim, Germany; CAS: 8042-47-5) to the assigned concentration (from 10^−2^ to 10^−6^). All odorants were prepared in 20 ml headspace vials, covered with nitrogen and sealed with a Teflon coated septum (Axel Semrau, Germany). Cancer samples were used in 1 ml aliquots and covered with nitrogen.

### Cell culture and cancer odour sample preparation

Cell culture and cancer odour sample preparation has been reported before^4^. Three different human breast cancer cell lines (SKBR3, BT474 and ZR75-1) and one non-transformed human mammary epithelial cell line (MCF-10A), referred as healthy control, were used in the experiments. The three human breast cancer cell lines were derived from different breast cancer histotypes: SKBR3 cell lines from metastatic breast adenocarcinoma (MetAC), BT474 cells from invasive ductal carcinoma (IDC) and ZR75-1 cells from metastatic invasive ductal carcinoma (MetIDC) (see ATCC.org website). These cancer cells were grown in DMEM culture medium (DMEM high-glucose medium (Sigma-Aldrich) supplemented with 10% fetal bovine serum (Sigma-Aldrich), 100 units/ml penicillin and 100 mg/ml streptomycin (Sigma-Aldrich). The immortalized, non-transformed human mammary epithelial cell line MCF-10A, was grown in DMEM/F12 medium (Sigma- Aldrich) supplemented with 5% fetal bovine serum, 20 ng/ml epidermal growth factor (EGF), 10 mg/ml insulin, 0.5 mg/ml hydrocortisone (Sigma-Aldrich), 100 units/ml penicillin and 100 mg/ml streptomycin (Sigma-Aldrich), as previously described^4,15^. All cells were cultured under standard conditions at 37 °C in humidified atmosphere containing 5% CO_2_.

For volatile organic compound (VOC) analysis, healthy control and the three cancer lines were seeded in triplicate in culture flasks (25 cm^2^) in 5 mL of their specific culture medium and were grown for 24 h. The number of plated cells was chosen based on the specific doubling time of every cell line, in order to obtain a comparable cell number at the end of the incubation of 24 h. After 24 h, the specific culture medium was removed and replaced with 5 mL of the DMEM culture medium. Cells were grown in these conditions for the next 96 h, up to a confluence of 50%-60% (around 1.5 ∗ 10^6^ cells/flask). After this incubation period, the DMEM culture medium was harvested, centrifuged at 1200 rpm for 5 min and collected in sterilized glass vials. Note that with this procedure, all samples derive from flasks with comparable cell density. The *control medium* was obtained by incubating DMEM culture medium in the same conditions as the cell samples, but without seeded cells.

### Calcium imaging

Calcium imaging was performed as described before^16,17^. In brief, we used a fluorescence microscope (BX50WI, Olympus, Tokyo, Japan) equipped with a 50x air lens (Olympus LM Plan FI 50x/0.5). A CCD camera (SensiCam, PCO, Kelheim, Germany) was mounted on the microscope recording with 8 × 8 pixel on-chip binning, which resulted in *p* = 80 × 60 pixel sized images. For each stimulus, recordings of 20 s at a rate of 4 Hz were performed using TILLvisION (TILL Photonics, Gräfelfing, Germany), resulting in *s* = 80 frames. A monochromator (Polychrome II or V, TILL Photonics, Gräfelfing, Germany) produced excitation light of 470 nm wavelength which was directed onto the antenna via a 500 nm low-pass filter and a 495 nm dichroic mirror. Emission light was filtered through a 505 nm high-pass emission filter.

### Stimulus application

Odorants were applied automatically, using a computer controlled autosampler (PAL, CTC Switzerland). 2 ml of headspace was injected in two 1 ml portions controlled by TTL pulses at time points 6 s and 9 s with an injection speed of 1 ml/s into a continuous flow of purified air flowing at 60 ml/min. The 1 s stimulus was directed onto the antenna of the fly via a Teflon tube (inner diameter 1 mm, length 38 cm). The autosampler syringe was flushed with purified air for 1 min after each injection and washed with pentane (Merck, Darmstadt, Germany), heated and flushed automatically for several minutes after each application of 1-butanol.

### Data processing

We extracted signals from the *in-vivo* calcium imaging movies with KNIME (www.knime.org) using the *ImageBee* plugin for insect imaging data^18^. We first corrected the imaging recordings for animal movement by rigid image registration with the *ImageBee* node *“Stabiliser”*. Each fluorescence value *f*_*j*_ at time point *j* = *1*..*s* was normalized by computing *Δf*_*j*_ */f* = *(f*_*j*_ *- f*_*0*_ *)/f*_*0*_, where *f*_*0*_ was the mean fluorescence value during 20 time points before stimulus application (time points from 3 to 22). Each calcium imaging recording can be represented by a *(m x p)* movie matrix *A*_*i*_ containing *m* odour features (*s* time points * odours) and *p* = *4*,*800* pixels (images were 80 × 60 = 4,800 pixels in size).

Thus, each of the *i* = *1*..*n* flies in the experiment contributed a (*m* x *p*) movie matrix *A*_*i*_ after concatenating all odorant response measurements. We employed an unsupervised feature selection approach to choose a subset of size *c* < *p* of the columns (pixels) of *A*_*i*_ that explained most of the norm of *A*_*i*_. We set *c* = *300* for antennal data based on previous data showing that using *c* = *300* could explain >99% of the norm of A_i_ for all recordings of a comparable data set (antenna imaging recordings with the same number of pixels)^4^. Thus, we selected *c* pixels from *A*_*i*_ into the (*m* x *c*) matrix *F*_*i*_ composed of c columns from *A*_*i*_, such that, when combined linearly with non-negative coefficients in the *(c x p)* matrix *X*_*i*_, the norm error ||*A*_*i*_ *−* *F*_*i*_ *X*_*i*_|| was minimized (Frobenius norm). Elements in the coefficient matrix *X*_*i*_ were constrained to be non-negative, so that columns inside the convex cone spanned by the columns in *F*_*i*_ could be reconstructed exactly, while the columns left outside of the cone contributed to the norm error. Minimization of the norm error criterion can be performed efficiently with the convex cone algorithm, as implemented in the *“ConvexCone”* node in the *ImageBee* plugin for KNIME. This identified a set of pixels whose signals varied most in response to the odorant stimulations. For noise reduction the selected pixels were replaced with means over spatially contiguous similar pixels^18^. For further details on data pre and post processing, see^4^.

We selected *t* = *10* time points corresponding to the two response peaks. Next, each time point was standardized (autoscaling). The (*t* x *c*) response matrix obtained for every odour was reshaped in order to obtain a *reference odour response spot vector* of dimensions (1 x *q*), with *q* = *t* ∗ *c*, representing the odorant response signal for each individual odorant. Next, we constructed a (*r* x *q*) *reference odorant projection matrix* for this animal, *P*_*i*_, by stacking the *reference odorant response vectors* gathered for *r* different reference odorants. Finally, we remapped the reference odorant stimulus space through

1 $${S}_{i}={({P}_{i}^{T})}^{+}=\,{P}_{i}^{T}\ast {({P}_{i}\ast {P}_{i}^{T})}^{-1}$$

with the plus (+) and *T* superscripts indicating the pseudoinverse and the transpose of *P*_*i*_, respectively. The matrix *S*_*i*_ represents the linear transformation that allows to set the reference odours of animal *i* as the basis of the new reference coordinate system. With this process, the way we imagine how a brain maps and subsequently categorizes odours is the same as how we would statistically cluster points in a multidimensional space. Here, we use an *r*-dimensional space, where *r* is the number of reference odorants used. Every (new) odorant response ***v***_*i*_^*local*^ for an odorant that is not a reference odour can now be expressed by an r-dimensional vector

2 $${v}_{i}^{global}={S}_{i}\ast {v}_{i}^{local}$$

Since this transformed vector ***v***_*i*_^*global*^ is in an animal-independent functional coordinate system, it is independent of the individual in which it was recorded, and can now be compared across individuals.

For every further animal *i*, each odorant response vector, ***v***_*i*_^*local*^, can be calibrated via its own animal-specific *S*_*i*_, into the global reference system ***v***_*i*_^*global*^, thus normalizing the space across animals.

### Pattern classification, cross-validation

The pattern recognition process for experiment 1 (Fig. 3A,B) was structured as follows: i) measurements from four animals were used as training set (highest and lowest concentration) and the measurements from the complementary three animals as test set (all concentrations); ii) two classifiers were used to derive a supervised model with an associated learning algorithm: either supporting vector machine (SVM) classifier, or a k-NN classification, a non-parametric, supervised, instance-based learning technique using the Manhattan distance as metric criterion^12^; iii) A confusion matrix was determined by comparing the known group labels and the predicted group labels. iv) Steps (i) to (iii) were repeated for all 35 possible combinations of 3 out of 7 animals, yielding a net result and an associated error. Pattern recognition procedures are susceptible to errors deriving from data preprocessing, or meta-parameter refining^19^. Therefore, we carefully excluded any preprocessing in our cross-validation procedure, and always used separate datasets for training and testing. We find that the different performance between odour sets (step iv) is an indication of the importance of the odours used, i.e. of how to span the reference odour space.

For experiment 2 (Fig. 3C), the pattern recognition process was structured similarly: i) measurements from seven out of ten animals were used as training set and measurements from the complementary three animals as test set; ii) the classification was performed using SVM or k-NN classification; iii) a confusion matrix was determined by comparing the known group labels and the predicted group labels. iv) Steps (i) to (iii) were repeated for all 120 combinations of 7 out of 10 animals. The SVM and k-NN algorithms were implemented through the statistical toolbox of MATLAB R2016a.

### Significance statement

Functional activity patterns are a fundamental property of brain function – from those representing sensory stimuli all the way to consciousness. Even when there are no landmarks and/or identified neurons, it should be possible to exploit common activity patterns as a reference space to map neural representations from one brain to the next. Here, we use the peripheral olfactory system of the fruit fly as a test system. We define a mathematical reference framework from known odorants, and map novel odour stimulus representations across individuals into this common sensory space. This allows to identify novel odours reliably, opening far reaching novel possibilities, and allowing for important insight into brain function. Furthermore, it will be a useful tool for practical developments, e.g. in using animals as highly efficient sensors, and/or for biotechnological developments.
